# An antibiotic-free antimicrobial combination of bacteriocins and a peptidoglycan hydrolase: *in vitro* and *in vivo* assessment of its efficacy

**DOI:** 10.1128/aem.02433-24

**Published:** 2025-06-24

**Authors:** Christian Kranjec, Thomas F. Oftedal, Kirill V. Ovchinnikov, Vinícius da Silva Duarte, Simen Hermansen, Magdalena Kaus-Drobek, Izabela Sabała, Davide Porcellato, Harald Carlsen, Morten Kjos

**Affiliations:** 1 Faculty of Chemistry, Biotechnology and Food Science, Norwegian University of Life Sciences 56625, Ås, Norway; 2 Department of Molecular Oncology, Institute for Cancer Research, Oslo University Hospital 155272, Oslo, Norway; 3 Mossakowski Medical Research Institute Polish Academy of Sciences 89216, Warsaw, Poland; Universidad de los Andes, Bogotá, Colombia

**Keywords:** antimicrobial agents, bacteriocins, combination treatment, bacterial biofilm, bacterial infections, animal infections, wound infection, antimicrobial resistance, *in vivo*

## Abstract

Mastitis is an inflammatory disease of the mammary gland usually caused by bacterial pathogens that gain physical access to the glandular epithelium through the teat canal. In bovines, common mastitis-causing agents are environmental or pathogenic bacterial species, including staphylococci, streptococci, enterococci, and gram-negative bacteria such as *Escherichia coli*. Current therapeutic strategies for bovine mastitis typically involve the administration of antibiotic formulations into the infected udder, which can result in increased selection for antibiotic resistance and the accumulation of antibiotic residues in milk. In this study, we sought to develop an antibiotic-free antimicrobial formulation for the treatment of bovine mastitis based on bacterial antimicrobial peptides (bacteriocins) and proteins (peptidoglycan hydrolases). Using a combination of *in vitro* assays with a range of bacteriocins, we show that the combination of the thiopeptide micrococcin P1 (MP1) and the lantibiotic nisin A (NisA) is a robust antimicrobial formulation that effectively inhibits the growth of bovine mastitis-derived bacteria, both in planktonic and biofilm-associated growth modes. The addition of AuresinePlus (Aur, a staphylococcus-specific peptidoglycan hydrolase) further increased the antimicrobial potency against *S. aureus*. Furthermore, using two mouse models, a skin infection model and a mastitis model, we show that the combination MP1-NisA-Aur effectively inhibits methicillin-resistant *S. aureus in vivo*. We discuss the potential and challenges of using antibiotic-free antimicrobial combinations in the treatment of bacterial infections.

**IMPORTANCE:**

The spread of antibiotic resistance is a major global concern. This is reflected in the One Health concept, which is based on the premise that the spread of antibiotic resistance can only be addressed through coordinated efforts to promote “healthy people, healthy environments, and healthy animals.” It is therefore of great importance to reduce the use of medically important antibiotics in agriculture, where treatment of bovine mastitis is one of the major drivers of antibiotics use. In this work, we investigate the use of antimicrobial peptides and proteins as an alternative treatment for bovine mastitis pathogens.

## INTRODUCTION

In the dairy cattle industry, mastitis is a serious concern for the welfare of the animals and an economic burden for farmers. It has been estimated that mastitis results in a gross margin loss of 11%–18% per cow per year, with 70% of the economic burden related to mammary tissue damage leading to reduced milk production (1, 2). Bacterial intramammary infections (IMIs), the main cause of bovine mastitis, can result from contagious mastitis-causing bacteria (i.e., *Staphylococcus aureus*, *Streptococcus agalactiae, Mycoplasma* spp.) or from environmental bacteria (i.e., coliform bacteria, *Enterococcus* spp., coagulase negative, non-*aureus* staphylococci, *Streptococcus uberis*) (3–7). In addition, some pathogens, such as *Streptococcus dysgalactiae*, can be transmitted through both these routes. Mastitis can be classified as clinical or subclinical, the former being characterized by overt signs of inflammatory disease and a reduction in milk production and quality. Subclinical mastitis is manifested by reduced milk production and increased somatic cell count in the milk without overt signs of disease, which is more common in older lactating animals (8–10). Subclinical mastitis also poses increased public health concerns linked to the consumption of dairy products contaminated by pathogenic agents or their thermostable toxins (11, 12).

Current mastitis treatments involve the use of antibiotics administered via intramammary infusion, intramuscular, or intravenous injections (13). Antibiotic therapy, however, poses serious concerns due to the high rate of therapeutic failure and accumulation of the antibiotic in the milk. Furthermore, due to the widespread use of antibiotics, infections are more frequently caused by antibiotic-resistant strains, rendering antibiotic treatments ineffective (14). In addition, some bacterial species, such as *S. aureus*, have a marked capacity to form biofilms, which strongly enhances their resilience to antibiotic therapy (15). The timing of initiating mastitis therapy is crucial; the best therapeutic results are obtained upon administration of the antimicrobial therapy during the dry period, the 6–8 weeks prior to calving where the cow is not milked (16, 17). Additionally, treatment of IMI during the dry period (dry cow therapy) prevents the production of nonsalable waste milk (18). Potential alternative antimicrobial therapies include the use of prophylactic vaccines, bacteriophages, bacterially derived antimicrobial agents such as bacteriocins, and bacteriolytic enzymes (19–22).

Bacteriocins, ribosomally synthesized bacterial peptides, are prime examples of metabolites with antimicrobial activity. It is thought that bacteriocins are produced by virtually all bacteria and, from an ecological point of view, are synthesized in order to confer a selective advantage to the producer in niche competition, as these molecules often display activity against closely related species (23–25). Bacteriocins generally kill target cells by disruption of the cell membrane (e.g., nisin [Nis], garvicin KS [GarKS], and enterocin EJ97) (26, 27), but there are also bacteriocins with intracellular targets, such as micrococcin P1 (MP1), which inhibits translation (28). Bacteriocins that are post-translationally modified, such as nisin and micrococcin P1, are typically regarded as belonging to class I; while bacteriocins that are unmodified, which include garvicin KS and enterocin EJ97, belong to class II (23). Nisin, micrococcin P1, and garvicin KS are characterized by broad-spectrum activity against gram-positive bacteria (29, 30). A truncated derivative of enterocin EJ97 called enterocin EJ97-short (EJs) is primarily active against enterococci and species of the genera *Streptococcus* and *Staphylococcus* but only weakly active against *S. aureus* (31, 32). In the last decades, bacteriocins produced by lactic acid bacteria (LAB) have received greater attention for their therapeutic potential (22). LAB are an essential part of many dairy products; therefore, LAB and the metabolites they produce are generally recognized as safe according to the Food and Drug Administration. With respect to this, the well-characterized LAB bacteriocins, nisin and pediocin PA-1, have been approved for use as food preservatives (25). It is also generally accepted that LAB bacteriocins have potential medical applications since they inhibit the growth of important human pathogens, including methicillin-resistant *S. aureus* (MRSA), vancomycin-resistant enterococci, and *Listeria monocytogenes*, and are effective against biofilm-forming strains (23, 33–38). Furthermore, since LAB are naturally present in raw dairy milk, they can potentially be used as probiotics to prevent and treat bovine mastitis (39–43).

Peptidoglycan hydrolases (PGHs), enzymes that catalytically degrade the peptidoglycan layer of the bacterial cell wall, have also been explored as potential alternatives to antibiotics (44–48). These hydrolytic enzymes can originate from bacteria (autolysins and exolysins) or bacteriophages (endolysins) (20, 47, 49). PGHs are often further modified to increase their potency, stability, or specificity by domain shuffling and/or site-directed mutagenesis. These modifications result in enzymes that are more effective under a broader range of conditions, which extends their potential applications (44, 45, 50). Notably, similar to bacteriocins from LAB, PGHs exhibit high target specificity for prokaryotic cells, which is thought to make them a safe option for antimicrobial treatment in human and veterinary medicine. Moreover, they display a low prevalence of resistance, a feature particularly relevant in the era of antimicrobial resistance (51). One such enzyme is AuresinePlus (Aur), a chimeric PGH created by fusing the catalytic domain of the LytM autolysin from *Staphylococcus aureus* with the SH3b cell wall-binding domain from lysostaphin (45). This enzyme exhibits precisely defined specificity and has been shown to selectively and effectively eliminate staphylococcal cells, including antibiotic-resistant strains such as MRSA. Notably, it also demonstrates high efficacy in biofilms, and eradication remains active in milk—a rare and highly desirable feature among enzybiotics (52).

In this study, we investigated the potential use of an antibiotic-free, bacteriocin-based combination against a broad collection of gram-positive bacterial strains associated with mastitis. We show that the combination of nisin A (NisA) and the thiopeptide bacteriocin micrococcin P1 serves as a scaffold to further build antibiotic-free antimicrobial combinations with increased potency and reduced occurrence of resistance. When combined with a PGH with high specificity to *Staphylococcus* spp., AuresinePlus, the combination demonstrated superior activity against biofilms formed by a panel of mastitis-derived staphylococci, including *S. aureus*. In addition, we show that the three-component combination effectively eradicates infections caused by methicillin-resistant *S. aureus* in two mouse models.

## RESULTS

### A combination of micrococcin P1, nisin A, and AuresinePlus is highly effective against a wide array of mastitis-derived strains

**Fig 1:**
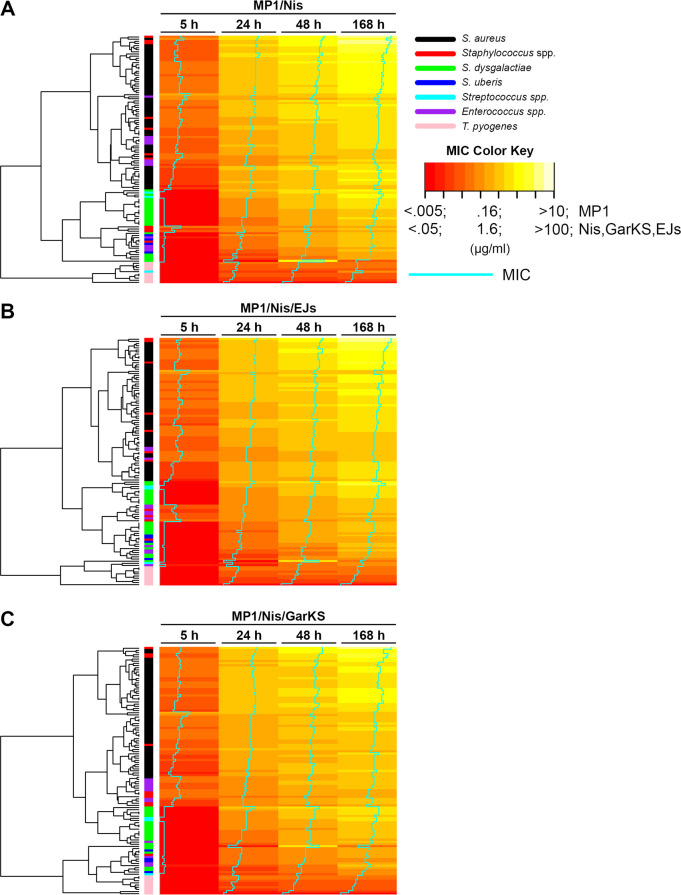
Heatmap showing the distribution of MIC values (µg/mL) for the indicated mastitis-derived species against the antimicrobial combinations (**A**) MP1/Nis, (**B**) MP1/Nis/EJs, and (**C**) MP1/Nis/GarKS. MIC values were measured 5, 24, 48, and 168 h after the addition of antimicrobial combinations. MIC values are indicated by color from red (low) to yellow-white (high) as indicated on the MIC color key and by a vertical line (in cyan) going from low (left) to high (right). The species of the strains tested (*n* = 116) are indicated by color (see legend). A dendrogram showing the clustering of strains based on their MIC values is shown to the left.

**Fig 2:**
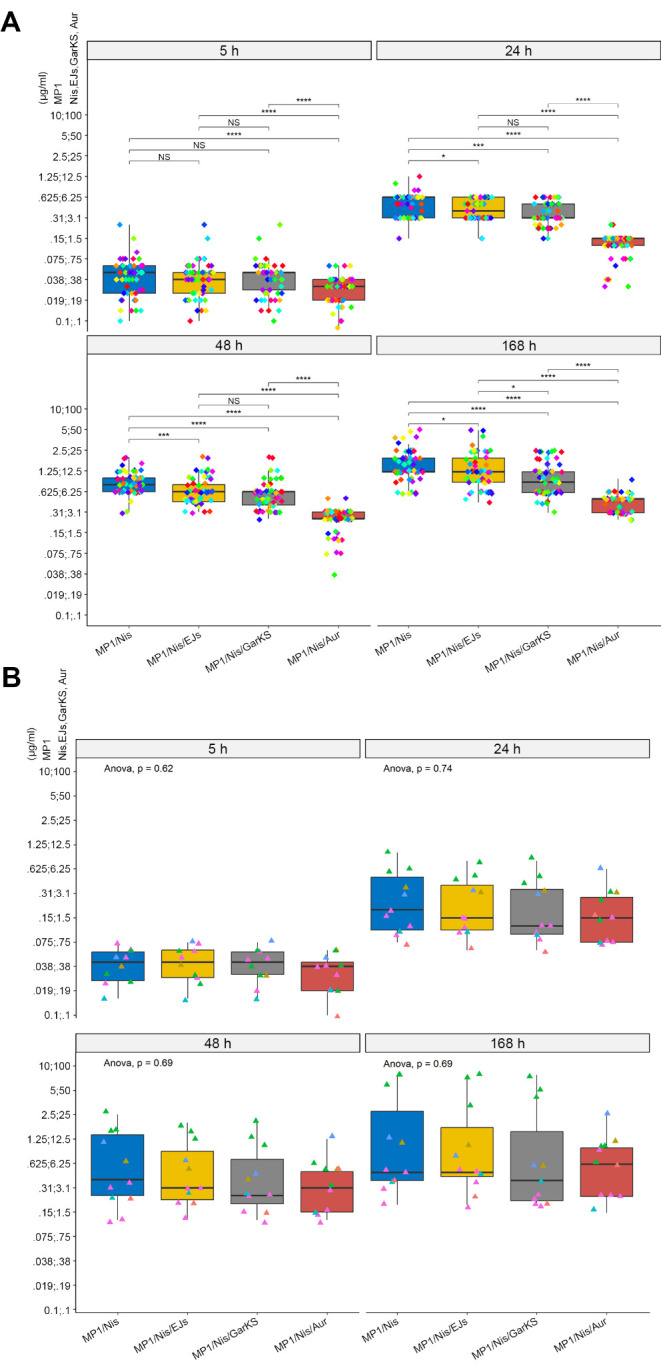
Aur potentiates the antimicrobial effect of bacteriocins against mastitis-derived *S. aureus*. (**A**) Box and whisker plot (Tukey’s method) showing the distribution in MIC for 57 strains of *S. aureus* included in our mastitis collection against the MP1/Nis, MP1/Nis/EJs, MP1/Nis/GarKS, or MP1/Nis/Aur combinations, measured 5, 24, 48, or 168 h after the addition of the antimicrobial combination. (**B**) Same as in panel A but against 11 strains of non-*aureus* staphylococcal species. In panel A, post hoc statistical analysis was performed as pairwise comparisons with the Wilcoxon test. Asterisk representation of statistical significance: *P ≤ 0.05; ***P ≤ 0.001; ****P ≤ 0.0001; NS = not significant. In panel B, the global statistical significance was assessed using the one-way ANOVA test (*P*-values indicated).

In this study, we assessed the activity of four bacteriocins with characterized spectra of activity: micrococcin P1, garvicin KS, EntEJ97-short, and nisin A against a pool of representative mastitis-derived bacterial species belonging to the genera *Staphylococcus*, *Streptococcus*, *Enterococcus,* and *Trueperella* (41, 53–57). Antimicrobials such as bacteriocins can significantly reduce bacterial growth but often fail to provide long-term inhibition due to resistance development, proteolytic degradation, persister cells, or other detoxification mechanisms in bacteria. As such, we assessed the ability of the combinations to provide long-term inhibition for up to 1 week (168 h). As shown in Fig. S1A and B, these assays identified bacteriocin combinations containing MP1 and Nis (MP1/Nis, MP1/Nis/EJs, or MP1/Nis/GarKS) as able to provide durable antimicrobial activity against planktonic mastitis-derived isolates in terms of low minimal inhibitory concentration (MIC). Next, we were interested in verifying the effectiveness of the MP1/Nis-containing antimicrobial combinations against a wider panel of 116 bovine mastitis-derived gram-positive isolates (Fig. S2A). The effectiveness of the antimicrobial combination was determined by measuring the minimum inhibitory concentration of planktonic cells at 5, 24, 48, and 168 h after the addition of antimicrobials to cultures. The results are presented in Fig. 1 and Fig. S2B. As can be seen in Fig. 1, while the MP1/Nis combination effectively inhibited planktonic growth of all strains, the addition of either EJs or GarKS resulted in a further reduction in MIC for all the bacterial strains (indicated by a shift in the blue vertical MIC line toward the left from panel A to B and C). Staphylococci (*S. aureus* and *Staphylococcus* spp.) were, however, more resilient, with some strains displaying consistently high MIC for all the treatment regimes. *Trueperella pyogenes* displayed the lowest MICs among the strains in the panel (Fig. 1; Fig. S2B). The addition of AuresinePlus to the MP1/Nis combination led to a highly significant decrease in the MIC for all *S. aureus* isolates, irrespective of the treatment duration, and reduced the interstrain variability within the *S. aureus* cohort (Fig. 2; Fig. S3). Conversely, in line with the known specificity of AuresinePlus, the contribution of the hydrolase against other staphylococcal species was less obvious, with only some of the isolates within the *Staphylococcus* spp. cohort (e.g., *S. simulans* and *S. haemolyticus*) being more susceptible to the hydrolase-containing combination (Fig. 2; Fig. S3) (45). Taken together, these results indicate that the antimicrobial spectrum of activity of the MP1/Nis/Aur combination covers the most important gram-positive mastitis-associated pathogens.

### Bacteriocins and AuresinePlus retain activity against biofilms of mastitis-derived bacteria

We next assessed whether our antimicrobial combinations could eradicate cells in biofilms produced by mastitis-associated bacteria since biofilm formation has been associated with the persistence of mastitis infections and potentially be responsible for cases of chronic mastitis (58, 59). By performing biofilm formation assays *in vitro*, we found that 92% of *Staphylococcus* strains and 58% of *Enterococcus* strains were good or strong biofilm formers (Fig. S4A through C), whereas the streptococcal isolates were mostly weak biofilm formers (Fig. S4D). Only weak or no biofilm formation was found among *T. pyogenes* isolates (data not shown).

**Fig 3:**
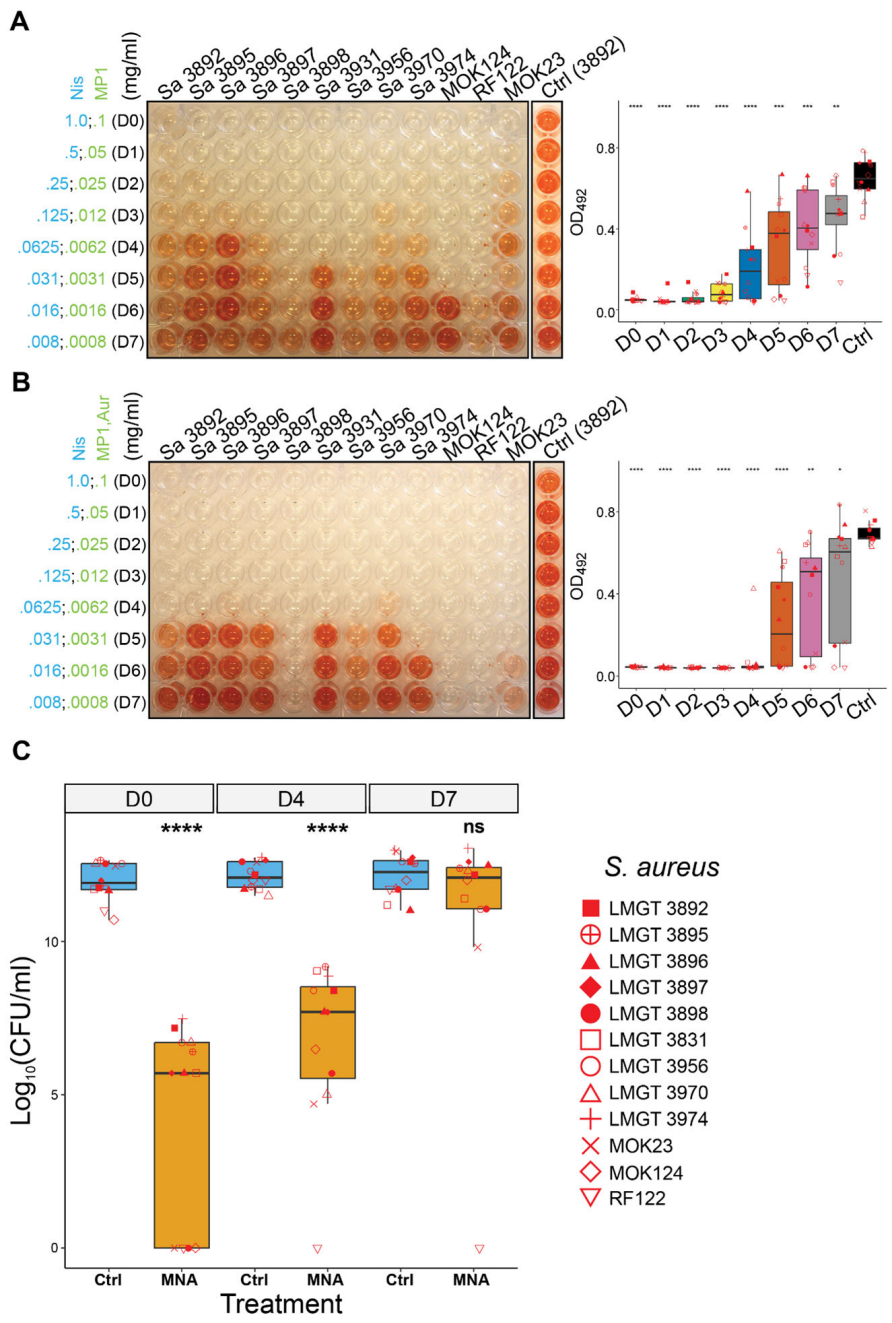
Assessment of bacteriocin and bacteriocin/AuresinePlus combinations against a panel of mastitis-derived *S. aureus* strains. (**A**) The left panel shows a representative image of the BOAT assay performed with a twofold dilution of the MP1/Nis combination against the indicated *S. aureus* strains. The concentrations of the antimicrobials (in mg/mL) are shown on the left (dilutions D0–D7). The assays were simultaneously performed with the control vehicles of the antimicrobials (Ctrl), and a representative image of *S. aureus* strain 3892 is shown. The amount of the red color is an indirect measure of metabolic activity and was measured by optical density readings at 492 nm (OD_492_). A plot of the quantification of red color (bacterial metabolic activity) as a function of the dilution factor is shown in the right panel. (**B**) Same as in panel A, except the strains were tested against the MP1/Nis/Aur combination. Statistical analyses of each group (D0–D7) relative to the vehicle control (Ctrl) were performed with Welch’s *t*-test. (**C**) Boxplot showing the median distribution of log10-transformed colony forming units (Log_10_CFU/mL) calculated after the BOAT assay for the same strains as in panels A and B. The CFU quantification was performed for the MP1/Nis/Aur combination (MNA) at concentrations D0, D4, and D7 depicted in panel B. Asterisk representation of statistical significance: *P ≤ 0.05; **P ≤ 0.01; ***P ≤ 0.001; ****P ≤ 0.0001; ns, not significant. Statistical plots (box and whisker) according to Tukey’s method.

A modified version of the biofilm-oriented antimicrobial test (BOAT) was used to test whether biofilms formed by *S. aureus*, *Staphylococcus* spp., and enterococci were sensitive to combinations of bacteriocins and PGH. In this assay, the metabolic activity indicator triphenyl tetrazolium chloride (TTC) is used to indicate metabolic activity (viability) of biofilm-associated cells after antimicrobial treatment (36, 38, 55, 60, 61). As can be seen in Fig. 3, both MP1/Nis and MP1/Nis/Aur combinations significantly reduced biofilm-associated metabolic activity in 12 mastitis-associated strains of *S. aureus* after 24 h (Fig. 3A and B). The concentrations required to reduce the biofilms varied between strains (Fig. 3A and B). Notably, the susceptibility of all strains to the antimicrobials in the biofilm was reduced (ranging from 2.5- to 80-fold) compared to planktonic cells. The addition of AuresinePlus to the combination significantly reduced metabolic activities compared to MP1/Nis alone (*P* < 0.05, Fig. 3A and B). When assessing cell viability by determining colony-forming units (CFU) (Fig. 3C), a very significant overall reduction could be observed for MP1/Nis/Aur-treated *S. aureus* biofilms (*P* < 0.0001; Fig. 3C). Although metabolic activity measurements indicated no or low cell viability in the biofilms after treatment at concentrations D0–D4, 7 out of 12 strains were shown to be viable after treatment at D0 by CFU counting (CFU/mL >10^5^) and 11 out of 12 at D4. Such discrepancy between CFU data and metabolic activity has also been observed previously (35). The highest sensitivity to the MP1/Nis/Aur combination was seen for strain RF112, for which neither metabolic activity nor cell viability could be detected even at the lowest concentration tested (Fig. 3). Among other mastitis-derived species, biofilm-associated enterococci were highly sensitive to the MP1/Nis combinations (Fig. S5A). Against non-*aureus* staphylococcal species, AuresinePlus extended the overall effective concentration window of the antimicrobial combination (Fig. S6A and B) and, consequently, reduced the overall cell viability (Fig. S6C).

Taken together, these data indicate that the bacteriocins and AuresinePlus make an effective antimicrobial combination active on biofilm-associated bacterial cells to reduce their viability.

### Effect of the combination of micrococcin P1, nisin A, and AuresinePlus against *S. aureus* in a murine skin infection model

To assess the therapeutic potential of the MP1/Nis/PGH combination *in vivo*, we utilized a murine skin infection model together with a bioluminescent methicillin-resistant *S. aureus* strain Xen31 (PerkinElmer, bioluminescent derivative of ATCC 33591). The skin infection model has previously been used and validated in our laboratory for the *in vivo* study of different antimicrobial combinations (34, 35). Furthermore, *S. aureus* Xen31 exhibits comparable susceptibility to the MP1/Nis/Aur combination in *in vitro* assays as the mastitis-associated strains used in this study (Fig. S7A and B).

**Fig 4:**
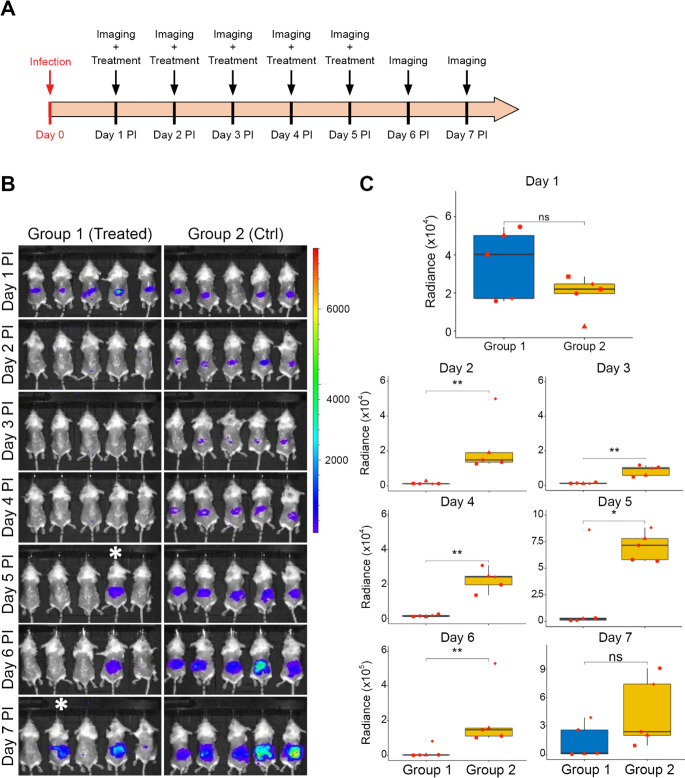
*In vivo* assessment of the efficacy of the combination of micrococcin P1, nisin A, and AuresinePlus (MNA) in a skin wound mouse model. The concentration of antimicrobials is the same as D0 in Fig. 3B (1 mg/ml nisin A, 0.1  mg/ml MP1, 0.1  mg/ml AuresinePlus) and the treatment was performed from day one post-infection to day 5 PI. Imaging and bioluminescence measurements from day 1 were taken prior to initiating treatment. (**A**) A schematic representation of the experimental setup. (**B**) *In vivo* imaging of the bioluminescent signals (in photons per square centimeter per steradian) produced by *S. aureus* Xen31 over the 7 day experiment in the treated group one and untreated group 2 (Ctrl). A relapse of the infection was seen in two mice of the treated group 1, indicated by white asterisks. (**C**) Quantification of the bioluminescent signals. Statistical significance between experimental groups was assessed with Welch’s *t*-test. Asterisk representation of statistical significance: *P ≤ 0.05; **P ≤ 0.01; ns = not significant.

Skin infections were established on the back skin of female BALB/c mice by inoculating 107 CFU of *S. aureus* Xen31 in freshly produced wounds of 10 mm in diameter. The infection was allowed to establish for 24 h prior to initiating antimicrobial treatment at day 1 post-infection (PI). Importantly, at this time point, infections had established, and the bacterially produced bioluminescence could be detected (Fig. S8). Mice were randomly divided into two groups: group 1 (MP1/Nis/Aur treated) and group 2 (vehicle-treated controls). Mice were then subjected to bioluminescence imaging daily from day 1 PI to day 7 PI, while treatment was administered daily from 1 through 5 PI (Fig. 4A). Bioluminescence imaging of mice in both groups prior to the initial treatment showed no significant difference between the two groups (Fig. 4A, *P* = 0.13). However, following treatment with the MP1/Nis/Aur combination (at concentrations equivalent to D0 in Fig. 3), we observed a substantial reduction in bioluminescence in all mice of group 1 (Fig. 4B). The bioluminescent signals in the MP1/Nis/Aur-treated group remained significantly lower than mice in control group 2 up to day 6 PI (Fig. 4C). However, from day 5 PI and onward, bioluminescence signals indicated a relapse of the infection in mice of group 1, one mouse by day 5 PI and two mice by day 7, indicating possible resistance development to the antimicrobial combination (Fig. 4B, white asterisks).

To further investigate this, bacteria were recovered from the wounds of the two mice (isolates Mutant 1 [Mut1] and Mutant 2 [Mut2]), and their susceptibility to MP1/Nis/Aur was reassessed by a spot-on-lawn assay. As shown in Fig. S9, a smaller zone of inhibition was seen for both isolates for the combination (MNA) compared to the wild-type strain Xen31. Inhibition zones were particularly reduced for the individual components MP1 and Nis, where one isolate (Mut2) appeared insensitive to MP1 at the concentration tested. Susceptibility to Aur appeared to be similar to the wild type for both isolates. To explain the resistant phenotype of the two isolates, whole-genome sequencing was performed to identify potential mutations (Table 1). Both isolates were found to differ from the wild-type strain only in the *rplK* gene, encoding the 50S ribosomal protein L11 (RefSeq accession YP_039991.1). Mut1 had a missense mutation causing an amino acid change from proline to serine (P26S), while Mut2 was found to have an in-frame duplication of 12 nucleotides causing an insertion of 4 amino acids (LeuGlyGlnAla) between amino acids at positions 31 and 32.

**TABLE 1:** Mutations found in *S. aureus* Xen31-derived isolates showing resistance toward the MP1/Nis/Aur antimicrobial combination in the wound and decreased sensitivity toward MP1 and Nis *in vitro^a^*

Strain	Mutation	Consequence on protein	Gene
Xen31 Mut1	c.76C > T	p.Pro26Ser	*rplK*
Xen31 Mut2	c.82_93dupTTAGGTCAAGCA	p.A31_G32insLGQA	*rplK*

^*a*^ c., chromosome; p., protein; dup, duplication; ins, insertion.

### Effect of the combination of micrococcin P1, nisin A, and AuresinePlus against *S. aureus* in a murine mastitis model

**Fig 5:**
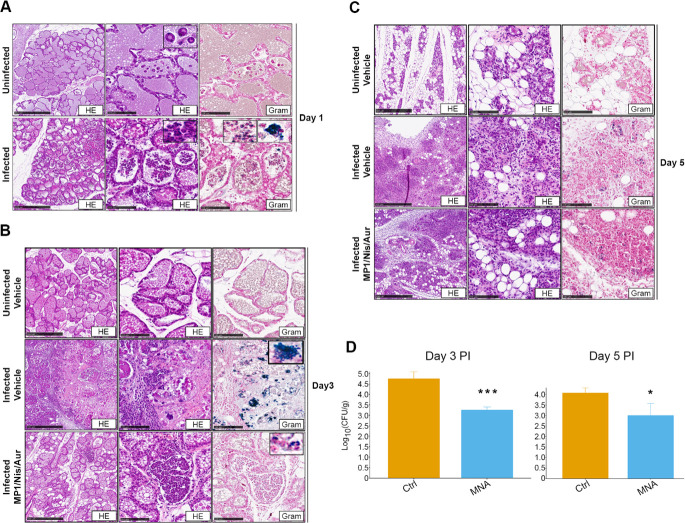
*In vivo* assessment of the efficacy of the combination of micrococcin P1, nisin A, and AuresinePlus in a mouse mastitis model. Representative images of the histological examination of explanted mammary glands. (**A**) from untreated *S. aureus* Xen31-infected and uninfected mice. (**B, C**) Uninfected mouse injected with the antimicrobial vehicle and infected mice treated with either the antimicrobial vehicle or the antimicrobial combination at the indicated time points. Note that the injections of the antimicrobials or their vehicles began at day 1 PI and were extended up to day 5 PI. The concentration used was the same as that corresponding to D0 in Fig. 3B (1 mg/mL nisin A, 0.1  mg/mL MP1, 0.1  mg/mL AuresinePlus). Sequential 5-µm-thick tissue sections were stained with either hematoxylin and eosin or Gram stain (Gram), and images were taken at 5× (left panels) or 20× (middle and right panels). Scale bars represent 500 µm in 5× images and 100 µm in 20× images. Insets show magnified images of areas of interest. (**D**) Barplots showing the log10-transformed average colony forming units (Log_10_CFU/g ± 1 standard deviation [error bars]) of bacteria recovered from homogenized mammary glands at day 3 PI (left) and day 5 PI (right). Statistical significance relative to the infected control (vehicle) was assessed with Welch’s *t*-test. Asterisk representation of statistical significance: *P ≤ 0.05; ***P ≤ 0.001.

Mastitis models take advantage of the conserved structure and function of the mammary gland epithelium across mammals and have proven valuable as a tool to investigate host-pathogen interactions within the glands (62, 63). Conventionally, lactating mice are used for this procedure, and the infection is induced by direct injection of bacteria or other mastitis-causing agents within the mammary gland through the teat canal. In order to develop the mastitis model in our laboratory, lactating CD-1 female mice were subjected to intramammary injection of 103 CFU of *S. aureus* Xen31 at 5–10 days after parturition. The infection was then monitored, with or without treatment, up to 7 days PI. As can be seen in Fig. 5A at 1 day PI, both the uninfected and infected mammary tissues show prominent lobular proliferation with dilated lobules containing eosinophilic material consistent with lactational secretions. The infected mammary tissues show acute immune reaction as evidenced by the presence of neutrophilic infiltrates within the lobular acini as visualized by histochemical staining (hematoxylin and eosin [HE]) (Fig. 5A). Gram staining of the same tissues highlighted gram-positive cells within the neutrophils (Fig. 5A, bottom-right panel). These cells appeared to reside predominantly either within phagocytic cells or as larger cell agglomerates resembling biofilm structures (64). At 3 days PI, after receiving the first two rounds of treatment (day 1 and 2 PI, see Materials and Methods), the vehicle-treated, uninfected mice (Fig. 5B, top panels) showed intact lobules and acini containing lactational secretions. Conversely, glands of vehicle-treated infected mice show necrosis of the acini and the lobular stroma, hemorrhage, and marked inflammatory neutrophilic infiltrates of the lobules (Fig. 5B, middle panels). Bacterial clumps were present in the lobular acini, which resembled biofilm-like structures within the infected tissue. In contrast, the glands of mice treated with the MP1/Nis/Aur combination showed neutrophilic infiltrates limited within the acini and fewer foci of gram-positive organisms. Furthermore, mice in the treated group had a reduced bacterial CFU count compared to vehicle-treated mice (Fig. 5B, bottom panels and Fig. 5D). By day 5 PI, all tissue samples displayed lobular involution with significant reduction of milk secretion in the acini. Lymphoplasmacytic infiltrates could be observed in the lobules of the infected mice, and intralobular fibrosis was more pronounced in the infected vehicle-treated mice than in the infected MP1/Nis/Aur-treated mice (Fig. 5C, middle and bottom panels, respectively). A significant reduction in bacterial counts (CFU) from isolated mammary tissues from mice treated with the Nis/MP1/Aur combination was also observed at day 5 PI, although the reduction was less significant compared to day 3 PI (Fig. 5D). By day 7 PI, no gram-positive cells could be detected in vehicle-treated or antimicrobial-treated glands, and both displayed a similar morphology to the uninfected vehicle-treated glands (Fig. S10A). Treatment of the mammary glands with the MP1/Nis/Aur combination, in the absence of bacterial infection, did not markedly alter the tissue as the appearance compared with the uninfected and untreated controls was similar for the respective time points (compare Fig. S10B).

## DISCUSSION

Mastitis is the most important disease impacting the dairy industry and the welfare of dairy cattle and is frequently caused by gram-positive bacteria, such as *Str. dysgalactiae*, *Str. uberis,* and *S. aureus* (65). In this study, we evaluated an antibiotic-free antimicrobial combination for combating mastitis-associated pathogens both *in vitro* and *in vivo*.

Initially, a combination of bacteriocins, micrococcin P1 and nisin, was assayed against a panel of mastitis-derived bacteria (Fig. 1A). This combination provided good inhibition against most species for a 5-h treatment with low MIC values *in vitro*, except for *S. aureus* which generally exhibited a lower susceptibility to the bacteriocins (higher MIC). However, at 24, 48, and 168 h post-treatment, inhibition was only observed at very high concentrations of the antimicrobials. To extend the effectiveness of the antimicrobial treatment beyond 5 h, the inclusion of enterocin EJ97s or garvicin KS was tested. Both three-component combinations provided improved inhibition, particularly at 48 and 168 h (Fig. 2A), but neither combination proved effective against staphylococci and *S. aureus* in particular. To overcome this limitation, we included AuresinePlus to the bacteriocin combination. This is an engineered peptidoglycan hydrolase with high potency and specificity against staphylococcal species such as *S. aureus* (45). AuresinePlus, together with the MP1/Nis combination, provided a significant reduction in MIC against all *S. aureus* isolates at all time points past 5 h compared with other combinations (Fig. 2A). The reduction in MIC for other staphylococcal species was also observed, but the effect was not as pronounced as for *S. aureus*, highlighting the specificity of AuresinePlus (Fig. 2B).

The antimicrobial combinations of MP1, Nis, and AuresinePlus were additionally shown to be effective at eradicating *S. aureus* biofilms (Fig. 3B and C). This led us to assess the antimicrobial efficacy of the combination *in vivo* using a murine skin infection model. For infected mice treated with the combination, a reduction in the bioluminescence signal was observed throughout the experiment, except for two mice where a relapse of the infection occurred (see Discussion below, Fig. 4C). Thus, our tripartite bacteriocin/PGH combination effectively inhibited the growth of *S. aureus* Xen31 *in vivo*. In the present study, we did not correlate bioluminescence with the amount of bacteria present in the wounds. Furthermore, we did not assess possible cytotoxicity of the combination and its effect on wound healing. However, similar studies have been performed on the individual components nisin and MP1, and the enzymes lysostaphin and ClyS, which have comparable structure and function to AuresinePlus (35, 38, 66–68). A study by van Staden et al. (69) reported that skin wounds infected with *S. aureus* Xen36 treated with nisin at a concentration comparable to that used in our study showed reduced bioluminescence and a 2-log reduction in bacterial counts with no negative effects on wound healing. Additionally, MP1 has previously been assessed for topical treatment of skin wounds in mice infected with *S. aureus* Xen31 as part of a formulation (0.1 mg/mL MP1, 5 mg/mL garvicin KS, and 5 mg/mL penicillin G in 5% hydropropylcellulose) (35), which also showed no obvious negative effects on the mice. Another engineered chimeric enzyme ClyS (chimeric lysin for staphylococci) incorporated into a topical ointment was shown to reduce *S. aureus* MW2 by 3-log (single dose of 10% [wt/wt]) in a murine skin infection model, with no macroscopic alterations to the wound (66).

A tripartite bacteriocin/PGH combination in which each of the components has a distinct mechanism of action, such as the ones used here, will not only be more effective due to its expanded antimicrobial spectrum, but it will also help counteract potential shortcomings of the individual components, such as instability and emergence of mutants. As individual components, these antimicrobials probably have limited efficacy against *S. aureus* in wounds. AuresinePlus is an engineered PGH consisting of the catalytic domain of the *S. aureus* autolysin LytM and the SH3b cell wall-binding domain lysostaphin from *S. simulans* (70). Lysostaphin belongs to a class of large (>10 kDa) heat-labile bacteriolysins; consequently, these enzymes are sensitive toward proteases. It is well known that wounds are protease-rich environments; the lower efficacy of AuresinePlus in the wound could therefore be due to the degradation of AuresinePlus in the wound (71). Furthermore, spontaneous mutations conferring high levels of nisin tolerance to *S. aureus* have been reported to occur at a frequency of 2 × 10^−7^ *in vitro* (72). Additionally, spontaneous mutants resistant to MP1 are frequently found *in vitro* but can also arise *in vivo* (35, 73, 74). Thus, while *S. aureus* can resist treatment with the individual components, it is highly improbable that the same *S. aureus* cells can evade all three components simultaneously.

Nevertheless, as mentioned above, two of the infected mice treated with the MP1/Nis/Aur combination showed an increase in bioluminescence after day 5 PI, suggesting a relapse of the infection. Suspecting that cells in these mice had developed resistance to one or more of the components in the treatment, whole-genome sequencing was performed on an isolate from each mouse. Both isolates harbored mutations in *rplK*, encoding the ribosomal protein L11. This was not unexpected, as the molecular target of MP1 is the *rplK*-encoded ribosomal protein L11, and certain mutations in this gene are known to confer resistance to MP1 and other thiopeptides (75). Binding of MP1 interferes with the interaction between ribosomal proteins L11 and L7, which inhibits a translocation step induced by hydrolysis of elongation factor G (75). Both mutants retained the susceptibility to AuresinePlus. Notably, however, the two isolated *rplK* mutants showed cross-resistance also to nisin. To our knowledge, mutations in *rplK* have not previously been shown to reduce susceptibility toward nisin. Nisin inhibits the growth of bacteria by binding to and sequestering lipid II, an essential precursor molecule for cell wall biosynthesis (76). Additionally, at higher concentrations, nisin in complex with lipid II can oligomerize into a pore-forming complex resulting in a loss of membrane potential and leakage of intracellular contents (27). It is uncertain how mutations in *rplK* confer tolerance toward nisin. It could be speculated that the reduced growth rate in these mutants resulting from lower translation efficiency can partially compensate for the inhibition of cell wall synthesis and lead to persistence. Mutations associated with nisin resistance have previously been shown to occur in genes encoding the two-component stress response system BraRS or the response regulator PmtR (77–79). It is not known if mutations in BraRS affect the fitness or virulence of *S. aureus*, but a mutant of *pmtR* showed reduced virulence and survivability in a mouse infection model, effectively limiting the problem with the emergence of such mutations *in vivo* (80).

Encouraged by the efficacy of the MP1/Nis/Aur combination in inhibiting *S. aureus* in the wound, we sought to examine the potential of the antimicrobial combination in treating mastitis, an infection often caused by *S. aureus*. Intramammary injection of the combination significantly reduced bacterial counts compared to untreated controls, clearly demonstrating the effectiveness of the tripartite combination in reducing the intramammary infection (Fig. 4C). Furthermore, intramammary injection of the combination in uninfected mice showed no considerable morphologic changes in the glandular architecture of the breast tissues (Fig. 5), suggesting low cytotoxicity of the combination at the concentration tested. Lysostaphin has been documented to be well tolerated by normal human epidermal keratinocytes with IC_50_ (midpoint cytotoxicity value) of 16 mg/mL (81). However, nisin and micrococcin P1 exhibit some cytotoxicity to eukaryotic cell lines in *in vitro* assays with IC_50_ of 0.3–0.4 mg/mL for nisin (HT29 and Caco-2 cell lines) (82). Micrococcin P1 impairs growth of HepG2 and THP-1 cell lines at concentrations above 0.03 mg/mL (28). Potential negative effects of the combination on the mammary tissue are likely reduced by the immediate diffusion and dilution of the components within the cells and interstitial fluid. However, we cannot exclude cytotoxic effects that are not easily apparent from histologic examination with HE stain.

Taken together, these assays demonstrate that the bacteriocin/PGH combination can effectively treat infections *in vivo*. However, for an optimal and long-lasting effect, proper antimicrobial dose and administration frequency are critical. It should be noted that while the tripartite formulation was highly potent, we did not systematically optimize the proportions of the antimicrobial components in the current work. Such optimization could potentially improve the effect of the combination treatment. We expect antimicrobial combinations to play an increasingly important role in combating multidrug-resistant bacteria. In contrast to monotherapeutic strategies, combinations of several antimicrobials have the potential for broader inhibition spectra and to reduce chances of resistance development. However, the development of resistance to alternative antimicrobials such as bacteriocins and the emergence of more general cross-resistance mechanisms will require more research. By employing antimicrobials with different non-overlapping mechanisms of action, resistance development becomes exceedingly unlikely. As with other drugs, further development of the treatment tested here will also require more knowledge about the cytotoxicity and the pharmacodynamics of the antimicrobial combination. Furthermore, by utilizing antibiotic-free antimicrobial agents when possible, we may hope to prolong the effectiveness of current antibiotics.

## MATERIALS AND METHODS

### Bacterial strains

All bovine mastitis-derived strains have been obtained from Tine Mastitis Laboratory (Molde, Norway) with the exception of *S. aureus* MOK strains and RF122, which were a kind gift from Orla Keane (Teagasc, Oak Park, Carlow, Ireland). All strains were routinely grown in brain heart infusion (BHI) broth (Oxoid, United Kingdom) at 37°C under aerobic conditions without shaking, with the exception of *T. pyogenes* strains, which were grown in BHI supplemented with 5% heat-inactivated fetal bovine serum (VWR). For *in vivo* imaging of bacterial infection in mice, *S. aureus* Xen31 (PerkinElmer, Waltham, MA) was used. The strain was derived from the parental strain *S. aureus* ATCC 33591, a clinical MRSA strain isolated from Elmhurst Hospital in New York, NY, USA (83). *S. aureus* Xen31 possesses a stable copy of the modified *Photorhabdus luminescens luxABCDE* operon at a single integration site on the bacterial chromosome.

### Antimicrobials

GarKS peptides and EJs were synthesized by Pepmic Co., Ltd. (China) with ≥95% purity and solubilized to a concentration of 1  mg/mL in sterile Milli-Q water. Micrococcin P1 was produced and purified as described previously (purity >98% as determined by high-performance liquid chromatography [HPLC] against a commercial standard of micrococcin P1, Cayman Chemicals) and solubilized to a concentration of 10 mg/mL in pure dimethyl sulfoxide (Sigma-Aldrich) (34). Nisin A was obtained as a commercial fermentate from Sigma-Aldrich (N5764; 2.5% [wt/wt] nisin, NaCl balanced with denatured milk proteins, and milk sugars), which was solubilized to a concentration of 1 mg/mL nisin (40 mg/mL of fermentate) in 0.05% (vol/vol) acetic acid solution (Merck). AuresinePlus was produced as recombinant protein as described previously (45) and stored in lyophilized form. AuresinePlus was solubilized to a stock concentration of 1 mg/mL in a buffer containing 20 mM Tris-HCl pH 7.0, 200 mM NaCl, 10% glycerol. All antimicrobials were diluted to their working concentrations on the day of the experiments. Stock solutions were stored at −20°C until use. Hydroxypropyl cellulose (HPC) with a weight-average molecular weight of ∼80,000 g/mol and a number-average molecular weight of ∼10,000 g/mol was used (Merck).

### Planktonic cell growth inhibition assays

Growth inhibition assays were performed in 96-well microtiter plates (Sarstedt). Briefly, 135 µL of growth medium was dispensed in each well of the plate (according to the number of bacterial strains tested), except in the wells of the first row. The antimicrobials were diluted in the growth medium to working concentrations (see below) in a final volume of 285 µL and dispensed in the wells of the first row. From the first row, 150 µL of the antimicrobials was then serially diluted (twofold dilutions) in a sequential fashion until the last row of the plate. Finally, 15 µL of a fresh overnight (O/N) culture of each strain was added in the appropriate wells to reach a final volume of 150 µL in each well. The plates were then incubated at 37°C for 5, 24, 48, or 168 h. The growth inhibition was expressed as a minimum inhibitory concentration, which refers to the minimum concentration of the antimicrobial needed to abolish the bacterial cell growth. The MIC values were assessed by optical density readings at 600 nm (OD600).

The growth media used for the assays were BHI for all the strains with the exception of *T. pyogenes* strains (see “Bacterial strains,” above). The working concentrations of the antimicrobials were as follows: 100 µg/mL for garvicin KS, EJs, nisin A, and AuresinePlus and 10 µg/mL for micrococcin P1.

### *In vitro* biofilm production and biofilm formation ability assay

Ten microliters of the O/N cultures was diluted 1/10 in 90 µL of the appropriate medium and dispensed in the wells of a 96-well microtiter plate (Sarstedt) to a final volume of 100 µL. The plates were then incubated at 37°C for 24  h. After the incubation, the presence of the biofilm at the bottom of the wells was initially confirmed visually. Biofilm formation ability assays were performed as described previously (36, 38). Bacterial biofilms were allowed to form for 24 h prior to being washed twice with 100 µL of 0.9% NaCl at room temperature to remove planktonic cells. Biofilms were then left to air dry for 15 min. After drying, 200 µl of a 0.4% solution of crystal violet (Merck) was added to each well and incubated for an additional 15 min. The dye was then removed, and the wells were washed three times with 200 µl of 0.9% NaCl; bound crystal violet was then extracted by incubating the wells with 100 µl 70% ethanol. The extraction procedure was repeated twice, and the combined crystal violet amount extracted was quantified by optical density measurements at 600 nm. The quantification of the crystal violet released from the biofilm is a surrogate measure of the number of bacterial cells forming the biofilm.

Media used for the biofilm formation were as follows: tryptic soy broth (TSB, Sigma Aldrich) supplemented with 1% glucose and 1% NaCl for all *S. aureus* strains; TSB supplemented with 1% glucose for all other species with the exception of *T. pyogenes* strains, for which BHI supplemented with 5% fetal bovine serum was used.

### Biofilm-oriented antimicrobial test

The BOAT assays were essentially performed as described previously (36, 38). Serial dilutions of the antimicrobials were prepared in challenge plates as follows: 175 µL of TSB was transferred in each row of a 96-well microtiter plate, except for the first row, according to the number of microbial strains tested. In the first row of the plate, the antimicrobials were diluted to their respective working concentrations in a final volume of 350 µL of TSB. From the first row, 175 µL of the antimicrobial dilutions was then transferred to the second row of the plate and further serially diluted to the bottom of the plate. The same procedure was followed to prepare the controls, except that instead of the antimicrobials, an equivalent volume of the respective vehicles was used. Unless otherwise stated, the starting concentrations of the antimicrobials for all experiments involving biofilms were 1 mg/mL nisin A, 0.1 mg/mL for micrococcin P1 and AuresinePlus, and 0.65 mg/mL for EJs.

Bacterial biofilms were produced as described above. After washing the biofilm twice with 100 µL of sterile saline, a total of 150 µL of the antimicrobial and control dilutions were transferred from the challenge plate to the corresponding wells of the biofilm plate. The challenged biofilms were then incubated for an additional 24 h at 37°C. After the challenge period, the antimicrobial dilutions were removed, and the biofilms were carefully washed three times with 150 µL of the sterile saline. A total of 100 µL of TSB supplemented with 0.025% of triphenyl tetrazolium chloride (Sigma-Aldrich) were then added to each well of the plate and further incubated at 37°C for 5  h. The results were then assessed by monitoring the development (or not) of formazan (red color), a measure of metabolic activity by the bacterial cells. The medium was then removed, and the red formazan was solubilized by adding 200 µL of ethanol:acetone (70:30) mixture to each well and incubated O/N. The amount of extracted dye, reflecting the degree of bacterial cell metabolic activity, was then quantified by spectrophotometric readings at 492 nm.

### Determination of the bacterial viability after BOAT

The procedure for the BOAT assay was repeated as described above, except that instead of adding the TTC solution, the antimicrobial-challenged cells were resuspended in TSB and then serially diluted in TSB buffer. Serial dilutions of the bacterial cells were then plated on BHI agar plates and incubated at 37°C for 24 h. The results were then assessed by direct counting of the developed colonies, and the CFU was determined.

### Mice

Two to four mice were housed per cage in individually ventilated cages (Innovive Inc., San Diego, CA) during the whole experiment and maintained on a 12-h light/12-h dark cycle with *ad libitum* access to water and a regular chow diet (RM1; SDS Diet, Essex, United Kingdom). Cages were equipped with chewing sticks and igloo houses with a running wheel and kept at a temperature of 25°C ± 1°C and relative humidity of 50% ± 5%. The mice were acclimatized in our mouse facilities for at least 1 week before the start of experiments.

### Mouse skin infection model

Before infection and treatment, the mice were anesthetized and shaved on the back and flanks. Anesthetics were an injectable cocktail (ZRF) containing Zoletil Forte (Virbac, Carros, France), Rompun (Bayer, Oslo, Norway), and Fentadon (Eurovet Animal Health, Bladel, The Netherlands). The cocktail (containing 3.3 mg Zoletil Forte, 0.5 mg Rompun, and 2.6 µg fentanyl per milliliter 0.9% NaCl) was administered by intraperitoneal injection (0.1 mL ZRF/10 g body weight) and shaved on the back and flanks with an electric razor. The remaining hair was removed with hair removal cream (Veet; Reckitt Benckiser, Slough, United Kingdom) according to the manufacturer’s instructions. The next day, the mice were again anesthetized with ZRF cocktail (0.1 mL/10 g body weight), and two skin wounds were made on the back of each mouse with a sterile biopsy punch 6 mm in diameter (dermal biopsy punch; Miltex Inc., Bethpage, NY). Prior to infection, overnight-grown *S. aureus* Xen31 cells were washed twice in sterile saline and then suspended in ice-cold phosphate-buffered saline (PBS) buffer. Each wound was inoculated with 10 µl of PBS containing 2 × 10^7^ CFU of *S. aureus* Xen31 cells using a pipette tip. After bacterial application, the mice were kept on a warm pad for 10–15 min to dry the inoculum, and the wounds were then covered with a 40- by 50-mm Tegaderm film (3M Medical Products, St. Paul, MN, USA). The mice were then left for 24 h for the infection to establish. The day after (day 1 post-infection), the mice were anesthetized with 2% isoflurane, and the luminescent signal was measured with an IVIS Lumina II (PerkinElmer; 2 min exposure time). The luminescent signal was quantified with Living Image software (PerkinElmer) from regions of interest around the wound and expressed as photons per second per square centimeter per steradian.

Mice were then split into two groups: group 1, mice were treated with the antimicrobial combination (1 mg/mL nisin A, 0.1 mg/mL MP1, and 0.1 mg/mL AuresinePlus) in a 5% (wt/vol) HPC hydrogel; group 2, mice were treated with the antimicrobial vehicles diluted in 5% (wt/vol) HPC as a negative control. Mice were treated daily by injecting the antimicrobials or the vehicle controls (50 µL) directly on the infected wounds through the Tegaderm film. The treatments were administered from day 1 to day 5 PI, whereas the bioluminescence imaging was performed daily for the whole course of the experiments (days 1–7 PI).

### Mouse mastitis model

Time-mated pregnant female mice, guaranteed at E15 on delivery date, were purchased from Janvier Labs (France). Four to six lactating females, 5–10 days after parturition, were anesthetized with a constant flow of 2% isoflurane. The abdominal area of the mice was disinfected with a 70% (vol/vol) ethanol solution, and the fifth pair of mammary glands was injected with either 50 µL of a *S. aureus* Xen31 suspension containing 10^3^ CFU in endotoxin-free PBS (VWR) or endotoxin-free PBS alone using a 50-µL Hamilton Neuros 700 Series Microliter Syringe (Hamilton) equipped with a removable 33G blunt-end needle. Twenty-four hours after injection, mice were divided into four groups: 1, bacterially injected and treated with antimicrobial combination; 2, bacterially injected and treated with vehicle control; 3, PBS injected and treated with antimicrobial combination; 4, PBS injected and treated with vehicle control. Treatments (antimicrobial combination or vehicle control) were administered once daily from day 1 to day 5 PI via intramammary injection. Starting at day 3 PI and every other day onward (days 3, 5, and 7), four mice per group were sacrificed by cervical dislocation and subjected to surgical removal of the fifth pair of mammary glands. At the same time, one uninjected lactating female was also sacrificed at the same time points to serve as a tissue morphology and gland involution control. For each mouse, one of the glands was placed in sterile, endotoxin-free PBS and one in a 4% solution of paraformaldehyde (PFA, Sigma Aldrich). Mammary glands stored in PBS were immediately subjected to tissue homogenization using a GentleMACS Dissociator equipped with GentleMACS M tubes (Miltenyi Biotech) following the manufacturer’s instructions. The resulting homogenates were serially diluted in BHI medium and plated on selective BHI-agar plates containing 50 µg/mL ampicillin (Merck). The plates were incubated overnight at 37°C to allow the growth of ampicillin-resistant colonies that were used for CFU determination. The glands in PFA were stored at 4°C overnight to allow full-tissue fixation followed by histological processing.

### Histology

After fixation in PFA, the tissues were embedded in paraffin, and 5 µm sections were obtained using a microtome. Sections were stained with HE, and high-resolution images were acquired using an automated slide scanner.

### Sequencing and sequence analysis

Sequencing and library preparation were performed by Novogene (Beijing, China) in paired-end mode (2 × 150 bp) to an average sequencing depth of approximately 600 for each sample. Sequencing reads obtained from *S. aureus* Xen31 were assembled using Unicycler v0.5.0 with the SPAdes genome assembler v3.15.5 (84, 85). Assembled contigs were annotated using prokka v1.14.6 with the provided database for genus *Staphylococcus* (86). Sequencing reads obtained from *S. aureus* Xen31 Mut1 and Mut2 were directly mapped against the annotated contigs for detection of variants using snippy v4.6.0 (87).

### Statistical analysis and data representation

The statistical analysis and graphical representations for all data were performed with R Studio (Version 2023.06.0+421) and R (version 4.3.2) software.

## SUPPLEMENTAL MATERIAL

Supplemental material aem.02433-24-s0001.docx Figures S1 to S10 and supplemental methods.

ASM does not own the copyrights to Supplemental Material that may be linked to, or accessed through, an article. The authors have granted ASM a non-exclusive, world-wide license to publish the Supplemental Material files. Please contact the corresponding author directly for reuse.
